# Preoperative diastolic function predicts the onset of left ventricular dysfunction following aortic valve replacement in high-risk patients with aortic stenosis

**DOI:** 10.1186/cc9040

**Published:** 2010-06-03

**Authors:** Marc Licker, Mustafa Cikirikcioglu, Cidgem Inan, Vanessa Cartier, Afksendyios Kalangos, Thomas Theologou, Tiziano Cassina, John Diaper

**Affiliations:** 1 Faculty of Medicine (University of Geneva) and Department of Anaesthesiology, Pharmacology and Intensive Care, University Hospital, rue Gabrielle-Perret-Gentil, CH-1211 Geneva 14, Switzerland; 2 Department of Cardiovascular Surgery, University Hospital, rue Gabrielle-Perret-Gentil, CH-1211 Geneva 14, Switzerland; 3 Departement of Anesthesia and Critical Care, Cardiocentro Ticino, via Tesserete 48, CH- 6900 Lugano, Switzerland

## Abstract

**Introduction:**

Left ventricular (LV) dysfunction frequently occurs after cardiac surgery, requiring inotropic treatment and/or mechanical circulatory support. In this study, we aimed to identify clinical, surgical and echocardiographic factors that are associated with LV dysfunction during weaning from cardiopulmonary bypass (CPB) in high-risk patients undergoing valve replacement for aortic stenosis.

**Methods:**

Perioperative data were prospectively collected in 108 surgical candidates with an expected operative mortality ≥9%. All anesthetic and surgical techniques were standardized. Reduced LV systolic function was defined by an ejection fraction <40%. Diastolic function of the LV was assessed using standard Doppler-derived parameters, tissue Doppler Imaging (TDI) and transmitral flow propagation velocity (Vp).

**Results:**

Doppler-derived pulmonary flow indices and TDI could not be obtained in 14 patients. In the remaining 94 patients, poor systolic LV was documented in 14% (n = 12) and diastolic dysfunction in 84% of patients (n = 89), all of whom had Vp <50 cm/s. During weaning from CPB, 38 patients (40%) required inotropic and/or mechanical circulatory support. By multivariate regression analysis, we identified three independent predictors of LV systolic dysfunction: age (Odds ratio [OR] = 1.11; 95% confidence interval (CI), 1.01 to 1.22), aortic clamping time (OR = 1.04; 95% CI, 1.00 to 1.08) and Vp (OR = 0.65; 95% CI, 0.52 to 0.81). Among echocardiographic measurements, Vp was found to be superior in terms of prognostic value and reliability. The best cut-off value for Vp to predict LV dysfunction was 40 cm/s (sensitivity of 72% and specificity 94%). Patients who experienced LV dysfunction presented higher in-hospital mortality (18.4% vs. 3.6% in patients without LV dysfunction, *P *= 0.044) and an increased incidence of serious cardiac events (81.6 vs. 28.6%, *P *< 0.001).

**Conclusions:**

This study provides the first evidence that, besides advanced age and prolonged myocardial ischemic time, LV diastolic dysfunction characterized by Vp ≤ 40 cm/sec identifies patients who will require cardiovascular support following valve replacement for aortic stenosis.

## Introduction

More than 200,000 aortic valve replacements are performed annually worldwide and this number will continue to increase with the aging population. Over the last two decades, the operative mortality rate has steadily declined from 10% to 4% along with improvements in surgical and anesthetic techniques [1-3]. However, left ventricular (LV) dysfunction requiring the administration of inotropic drugs often occurs after separation from cardiopulmonary bypass (CPB) and has been associated with prolonged ICU and hospital stay [3,4]. Although this *myocardial stunning *usually resolves within 48 hours, it may lead to low cardiac output syndrome that has become the leading cause of postoperative death [5,6].

In large cohorts of patients undergoing cardiac surgery, post-CPB LV dysfunction has been linked to age, female gender, history of heart failure, recent myocardial infarct, low LV ejection fraction, prolonged aortic cross-clamping and complexity of surgery [7-11]. More recently, echocardiographic markers of preoperative LV diastolic dysfunction have been associated with difficulties in weaning patients from CPB [12,13].

Although clinical signs (for example, pulmonary congestion, New York Heart Association [NYHA] classes) and markers of systolic LV function (for example, LV ejection fraction) have been studied extensively and incorporated in scoring algorithms for predicting perioperative risk, the prognostic value of diastolic dysfunction assessed by transoesophageal echocardiography (TEE) has not been examined in patients undergoing aortic valve replacement [1,2,5,14,15]. Besides pulsed-wave Doppler measurements of mitral inflow and pulmonary venous flow, evaluation of diastolic function has recently been improved with color M-mode transmitral flow propagation velocity (Vp) and mitral valve annular velocities recorded by tissue Doppler imaging (TDI) [16,17].

The main purpose of this study was to identify predictors of LV dysfunction in high-risk patients with aortic stenosis undergoing valvular replacement. Secondarily, we analyzed different Doppler parameters of diastolic function regarding their ability to predict post-CPB LV dysfunction.

## Materials and methods

### Study design and settings

This prospective cohort study was conducted in a tertiary reference center, from January 2006 to December 2008. The study was approved by the Institutional Research Board of the University Hospital of Geneva and informed consent was obtained from each patient with severe aortic valvular stenosis who met the eligibility criteria. The Bernstein-Parsonnet algorithm was used to assess the operative risk of mortality [18]. During the study period, 108 patients were selected among a cohort of 145 surgical candidates undergoing elective aortic valve replacement, either isolated or combined with coronary artery bypass grafting or aortic root replacement. A predicted risk of mortality exceeding 9% was considered as an entry criteria. Exclusion criteria consisted of atrial fibrillation or flutter, implanted pacemaker, severe mitral stenosis or regurgitation, severe pulmonary hypertension (mean pulmonary artery pressure ≥45 mmHg), moderate-to-severe valvular aortic insufficiency and preoperative inotropic or ventilatory support. Patients were secondarily excluded if poor image quality precluded echocardiographic measurements. All patients were operated on by one of three board certified cardiac surgeons and were managed by the same team of cardiothoracic anesthesiologists.

### Perioperative patient management

The usual medications were continued on the morning of the procedure, except diuretics and angiotensin-converting enzyme inhibitors or angiotensin II antagonists that were interrupted one day before. In the operating theatre, all patients were equipped with a noninvasive oscillometric monitor (brachial artery pressure), a radial arterial catheter, a central venous line and a bispectral monitor of the electroencephalogram (BIS Aspect Medical Systems A-2000 XP, Newton, Maryland, USA). Anesthesia management consisted of intra-thecal morphine, low doses of intravenous sufentanyl and an infusion of propofol to target BIS values between 40 and 60. Cardiac preconditioning was also provided with inhaled isoflurane (1% to 1.5%) before CPB.

A TEE probe (T6210 Omniplane II Philips Medical System, Andover, MA, USA) was introduced after anesthesia induction and images were digitally acquired before CPB and stored on a Philips Sonos 5500 Ultrasound Imaging system (Philips Medical Systems).

After full heparinization, normothermic CPB was instituted with a nonpulsatile flow (2.2 to 2.5 L/minute/m^2^) and alpha-stat control for acid-base management. The circuit and the membrane oxygenator were primed with 2 L of normal saline solution and mean arterial pressure (MAP) was maintained between 50 and 70 mmHg with vasoactive medications as necessary. During aortic cross-clamping, myocardial protection was achieved by intermittent antegrade infusion of cold blood. The aortic valve prosthesis (Carpentier-Edwards Perimount; Jt. Jude Medical Inc. Minneapolis, Minnesota, USA) was implanted in the supra-annular position with interrupted mattress sutures. All patients received tranexamic acid (20 mg/kg) and the transfusion threshold was a hematocrit less than 18 to 20% during CPB and less than 25% before/after CPB.

**Figure 1:**
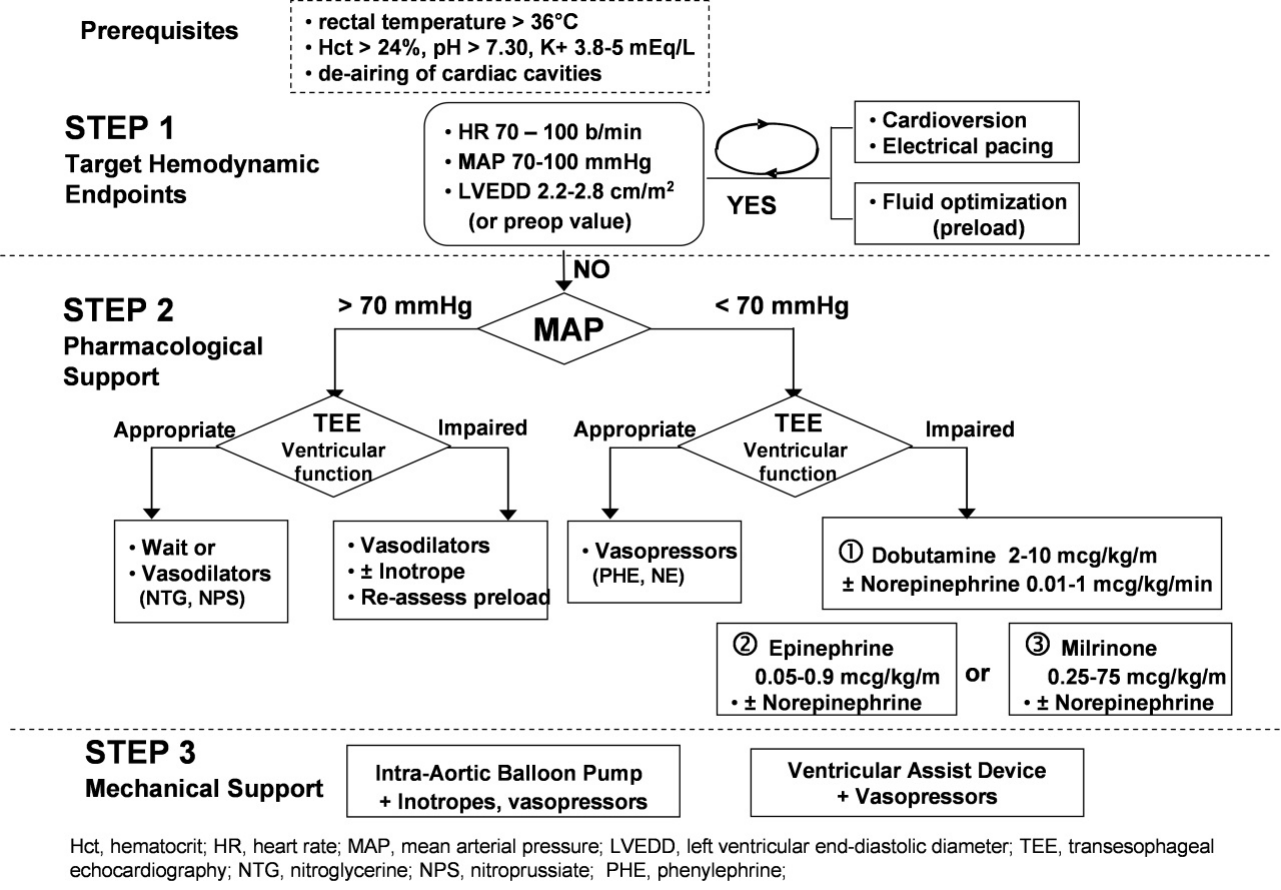
**Weaning protocol from Cardio-Pulmonary-Bypass**.

At the end of the procedure, weaning from CPB was guided by TEE assessment and hemodynamic measurements. After de-airing the cardiac cavities and resumption of mechanical ventilation, the pump flow was gradually reduced allowing filling of the cardiac chambers. In addition to fluid loading, electrical atrio-ventricular pacing, vasopressors and inotropes drugs as well as intra-aortic balloon pump (IABP) were eventually introduced to target the specific hemodynamic endoints: LV end-diastolic diameter (up to preoperative values or 2.2 and 2.8 cm/m^2^), MAP between 65 and 100 mmHg and heart rate between 70 and 100 beats/minute (see Figure 1).

The investigators performing the TEE were not involved in any therapeutic decision during the weaning process and the attending anesthesiologist in charge of the patient was blinded to the diastolic measurements. Pulmonary artery catheters were inserted in patients receiving inotropic support at the admission on the Intensive Care Unit (ICU).

### Study endpoints

The diagnostic criteria for post-CPB LV dysfunction was based on the need of inotropic support for at least two hours (dobutamine ≥5 mcg/kg/min, epinephrine >0.05 mcg/kg/min, milrinone >0.25 mcg/kg/min, norepinephrine >0.02 mcg/kg/min) in the presence of low MAP (<60 mmHg ascertained by both invasive and noninvasive pressure monitors) and with persistent, new or worsening LV functional impairment (for example, FAC (fractional area change) <40%). Secondary outcome variables were any postoperative cardiac adverse event occurring in the ICU such as myocardial infarct (troponin-I ≥1.5 ng/ml associated with new Q waves or ST segment abnormalities on the ECG, or with coronary artery intervention), supra-ventricular or ventricular arrhythmias (requiring anti-arrhythmic drugs or electrical cardioversion) and low cardiac output syndrome (cardiac index <2.2 L/min/m^2^, need for inotropic and/or IABP support to maintain MAP >65 mmHg).

### Measurements

During primary hospitalization, data related to patient demographic information, comorbidities, current medications, intraoperative TEE examination, indexed effective orifice area [19], anesthetic and surgical management as well as postoperative cardiac outcome were prospectively collected on a case report form and entered in a dedicated database.

A comprehensive TEE examination was performed before CPB using two-dimensional, M-mode, pulsed Doppler and TDI to assess systolic and diastolic LV function. In the transgastric short axis view, posterior wall thickness (PWT), LV end-diastolic and end-systolic areas (EDA and ESA, respectively) were measured. FAC of the LV was computed as (LVEDA -LVESA)/LVEDA. From a mid-esophageal four-chamber view, peak early (E) and late (A) mitral inflow velocities, deceleration time (DT) and isovolumic relaxation time (IVRT) were derived from recordings obtained with the pulsed Doppler sample volume positioned at the tip of the mitral leaflets. Peak systolic (S), diastolic (D) and atrial reversal velocities (Ar) were measured with the pulsed Doppler sample volume positioned within 1 to 2 cm of the left upper pulmonary vein. Thereafter, the TDI function was activated for recording early and late diastolic velocities of the mitral annulus (E' and A', respectively) by positioning the 5-mm sample volume within the septal and lateral insertion sites of the mitral leaflets to cover the longitudinal excursion of the mitral annulus. Finally, a color M-mode map was displayed from a mid-oesophageal four-chamber view, to obtain the longest column of flow from the mitral annulus to the apex. The M-mode cursor was aligned through the center, parallel to the transmitral inflow and a clear propagation wave front was obtained by adjusting the Nyquist limit and baseline shift. Vp was defined as the slope of the first aliasing velocity during early filling, measured from the mitral valve opening to 4 cm into the LV cavity.

Cardiac stroke volume was calculated as the flow surface area multiplied by the velocity time integral through the LV outflow tract obtained by pulsed wave Doppler. Cardiac index (CI) was calculated as the product of SV and HR divided by body surface area. All recorded values were averaged from three consecutive beats.

Poor systolic LV function was considered if the LV ejection was <40% on the preoperative transthoracic echocardiographic examination. According to the working group of the European Association of Echocardiography and the American Society of Echocardiography, LV diastolic function was graded into four classes: normal (E/A > 0.8, DT < 200 ms, and E'/A' > 1 or S/D 1 to 1.5), impaired relaxation (E/A < 0.8, DT > 200 ms, IVRT ≥ 100 ms and E'/A' <1 or S/D >1.5), pseudo-normalization (E/A = 1 to 2, DT = 150 to 200 ms, and E'/A' <1 or S/D <1.2), and restrictive pattern (E/A >2, DT <150 ms and E'/A' <1 or S/D <0.8) [19].

To test the intra- and interobserver variabilities, E and A, E' and A' as well as Vp were measured twice by two independent operators, in 10 randomly selected cases.

### Statistical analysis

Perioperative clinical, surgical and echocardiographic characteristics of patients with and without post-CPB LV dysfunction were compared with the χ^2 ^test for categorical variables (expressed in percentage) and the Student *t *test (normal distribution) or Wilcoxon rank test (non-Gaussian distribution) for continuous variables (all expressed as mean ± SD).

Variables that had a univariate probability value <0.20 or those judged to be clinically important were selected for inclusion in a logistic regression model by stepwise selection. To avoid multi-colinearity, only one variable was retained in a set of variables with a correlation coefficient greater than 0.5. Independent predictors of LV dysfunction and factor-adjusted odds ratios (ORs) with 95% confidence interval (CI) were calculated. Model discrimination was evaluated by the area under the receiver-operator-characteristic (ROC) curve, and calibration was assessed with the Hosmer-Lemeshow goodness-of-fit statistic. Receiver operating characteristics (ROC) curves were constructed to determine the best cut-off of echocardiographic parameters (with *P *< 0.2) to predict post-CPB LV dysfunction. All analyses were performed using SPSS software (version 14.0 for Microsoft Windows; SPSS, Chicago, IL, USA) and statistical significance was specified as a two-tailed type I error (*P *value) set below the 0.05 level.

## Results

Over a three-year period, 108 high-risk patients underwent valve replacement for severe aortic stenosis and 14 were excluded since Doppler-derived pulmonary flow indices and TDI could not be obtained (9 and 11 patients, respectively). In the remaining 94 patients, all presented LV hypertrophy (PWT >11 mm), poor systolic LV function was found in 14% of patients (n = 12) whereas diastolic dysfunction was diagnosed in 84% of patients (n = 89), all of whom had Vp < 50 cm/s. Regarding echocardiographic measurements, intra-and interobserver variabilities were lowest for Vp and highest for E' and A' measurements (Table 1).

**Table 1:** Intra- and interobserver characteristics

Characteristics	Intra-observer variability	Inter-observer variability
**E wave**	7.8	8.7
**A wave**	8.1	9.4
**Deceleration time**	8.9	9.6
**PV S**	7.1	8.2
**PV D**	8.3	9.1
**E' wave lateral**	7.9	8.8
**A' wave lateral**	8.1	9.2
**E' wave septal**	9.4	11.6
**A' wave septal**	9.7	10.9
**Vp**	3.2	4.8

PV S, peak systolic velocity of pulmonary venous flow; PV D, peak diastolic velocity of pulmonary venous flow; E, peak early mitral inflow; A, late mitral inflow; DT, deceleration time; E' and A', early and late diastolic annular velocities, Vp, transmitral flow velocity

During weaning from CPB, LV dysfunction occurred in 38 patients (40.4%). Inotropic support consisted in the administration of dobutamine (5.6 ± 2.7 mcg/kg/h, over 10 ± 5 hours), epinephrine (0.52 ± 0.41 mcg/kg/h over 6 ± 3 hours), norepinephrine (0.08 ± 0.04 mcg/kg/h over 12 ± 7 hours) and/or milrinone (0.27 ± 0.14 mcg/kg/h over 6 ± 2 hours). Five patients were also treated with an IABP in combination with inotropes. As shown in Table 2, MAP and CI were significantly lower in patients with post-CPB LV dysfunction. Of the 31 preoperative and intraoperative variables subjected to univariate analysis, eight demonstrated a significant association with the occurrence of post-CPB LV dysfunction (Table 3). Patients with post-CPB LV dysfunction were significantly older, they had lower LV ejection fraction, more severe grades of LV diastolic dysfunction, lower Vp as well as prolonged duration of CPB and aortic clamping.

**Table 2:** Hemodynamic in patients with and without post-CPB left ventricular dysfunction

	No LV dysfunction (n = 56)	With LV dysfunction (n = 38)	*P *value
**Mean Arterial Pressure, mmHg**			
Before CPB (10 minutes)	93 (12)	90 (15)	0.847
After CPB (10 minutes)	82 (14)	68 (16)*	0.012
**Heart Rate, b/min**			
Before CPB (10 minutes)	70 (8)	73 (9)	0.912
After CPB (10 minutes)	78 (13)	82(14)	0.634
**Central Venous Pressure, cm H_2_O**			
Before CPB (10 minutes)	6 (3)	7 (4)	0.879
After CPB (10 minutes)	8 (4)	9 (5)	0.953
**Cardiac Index, L/min/m^2^**			
Before CPB (10 minutes)	2.4 (1.0)	2.2 (1.3)	0.597
After CPB (10 minutes)	3.6 (1.3)	2.1 (0.9)*	< 0.001

LV, left ventricular; CPB, cardiopulmonary bypass.

*P < 0.05, between the two groups; χ2 test with Yates correction or unpaired Student *t *test.

**Table 3:** Distribution of perioperative variables according to the presence of post-CPB left ventricular dysfunction

	No LV dysfunction (n = 56)	With LV dysfunction (n = 38)	*P *value
***Preoperative clinical and biological variables***			
**Age (y)**	66 (9)	74 (10)	0.004
**Male Gender (%)**	61.8	66.7	0.288
**Body Mass Index (kg/m^2^)**	28 (5)	27 (6)	0.281
**Hypertension (%)**	85.1	82.3	0.654
**Diabetes Mellitus (%)**	30.9	27.5	0.745
**Coronary Artery Disease (%)**	29.1	38.5	0.412
**Peripheral Vascular Disease (%)**	12.1	10.9	0.511
**Dyslipemia (%)**	71.8	54.5	0.098
**Renal Insufficiency^1 ^(%)**	3.6	5.2	0.532
**Anemia^2 ^(%)**	7.1	10.1	0.789
**Beta-blockers (%)**	49.1	61.5	0.295
**ACE Inhibitors or AII antagonists (%)**	20.1	35.9	0.352
**Calcium channel antagonists (%)**	23.6	33.3	0.101
**Diuretics (%)**	21.9	17.9	0.796
**Nitrate (%)**	12.7	17.9	0.562
**LV Ejection Fraction (%)**	56 (8)	49 (11)	0.001
**Parsonnet score (u)**	15 (9)	21 (9)	0.001
			
***Intraoperative echocardiographic data***			
**LV Mass Index (g/m^2^)**	135 (34)	140 (29)	0.188
**LV Fractional Area Changes (%)**	54 (14)	46 (14)	0.015
**LV Septal Thickness (mm)**	16 (3)	17 (4)	0.899
**Transmitral E/A ratio**	1.2 (0.6)	1.3 (0.7)	0.415
**Deceleration Time (ms)**	181 (81)	158 (65)	0.173
**Isovolemic Relaxation Time (ms)**	108 (30)	106 (32)	0.897
**PV S/D ratio**	1.2 (0.4)	1.1 (0.5)	0.067
**PV Ar (ms)**	14 (9)	16 (7)	0.198
**E'/A' lateral**	1.0 (0.5)	0.9 (0.5)	0.736
**E'/A' septal**	1.0 (0.4)	1.0 (0.6)	0.716
**Vp (cm/s)**	53 (11)	37 (7)	<0.001
**Diastolic Functional Class**			<0.001
**Normal (%)**	26.8	0	
**Impaired Relaxation (%)**	50.0	34.2	
**Pseudo-normalization (%)**	23.2	42.1	
**Restrictive pattern (%)**	0.0	23.7	
			
***Surgical variables***	11	16	
**Type of Procedure (%)**			0.280
**Isolated Valve Replacement**	65.9	51.1	
**Associated Coronary Artery Bypass**	21.6	34.1	
**Associated Aortic Root Replacement**	12.5	14.8	
**Aortic Clamping Time (minutes)**	77 (28)	98 (40)	0.010
**Cardiopulmonary Bypass Time (minutes)**	101 (33)	135 (57)	0.004
**Indexed Effective Orifice Area**	1.05 (0.11)	0.98 (0.13)	0.634

LV, left ventricular; CPB, cardiopulmonary bypass.

*P < 0.05, between the two groups; χ^2 ^test with Yates correction or unpaired Student *t *test.

LV, left ventricule; ACE, angiotensin converting enzyme; AII, angiotensin II; PV Ar, pulmonary vein atrial reversal velocity; PV S/D, ratio of peak systolic to diastolic pulmonary vein flow velocities; E'/A', ratio of early to late diastolic velocities of the mitral annulus determined by tissue Doppler imaging.

^1^Renal insufficency defined as creatinine clearance <10 ml/minute.

^2^Anemia defined as Hemoglobin <110 g/L in female and <120 g/L in male.

Stepwise logistic regression analyses identified three independent predictors of LV dysfunction: age (OR = 1.11; 95% CI, 1.01 to 1.22), aortic clamping time (OR = 1.04; 95% CI, 1.00 to 1.08) and Vp (OR = 0.65; 95% CI, 0.52 to 0.81). This multivariate model for predicting LV dysfunction was robust, with an area under the ROC curve of 0.96 (95% CI, 0.89 to 0.99) and a Hosmer-Lemeshow goodness-of-fit probability value of 0.49 indicating good model calibration and discrimination. Substitution of aortic clamping time for CPB time and diastolic classes (1 to 4) for Vp, did not improve the area under the ROC curve. There was no evidence that additional covariates would improve the model (*P *= 0.21 by the Wald link specification test).

**Figure 2:**
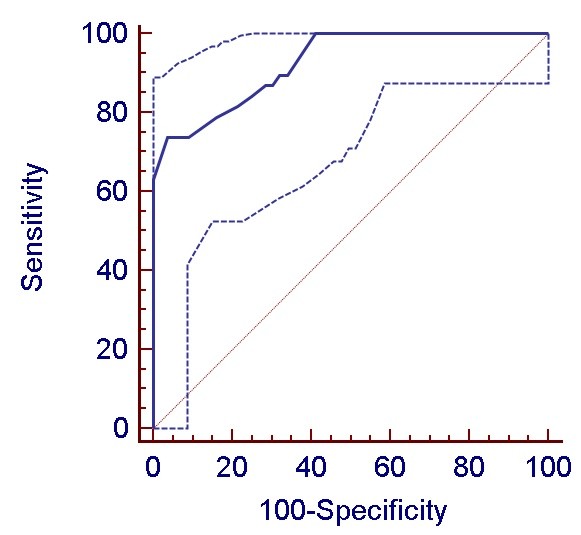
**Receiver operating characteristic (ROC) curves assessing the association of transmitral propagation velocities (Vp) with post-cardiopulmonary bypass left ventricular dysfunction (mean and 95% coinfidence limits)**.

As shown in Figure 2, the best cut-off value for Vp to predict LV dysfunction was 40 cm/s as it maximized both sensitivity (73%; 95% CI, 55% to 87%) and specificity (96%; 95% CI, 87% to 99%).

The expected mortality of the whole cohort was 22% whereas the observed mortality was only 10.6%. As shown in Table 4, compared with patients without post-CPB LV dysfunction, those experiencing LV dysfunction presented higher in-hospital mortality (18.4% vs. 3.6%, *P *= 0.044) and an increased incidence of serious cardiac events (81.6 vs. 28.6%, *P *< 0.001). These patients also required prolonged mechanical ventilation and longer stay in the ICU and in the hospital.

**Table 4:** Postoperative clinical outcome

	No post-CPB LV dysfunction (n = 56)	With post-CPB LV dysfunction (n = 38)	*P *value
**In-Hospital Mortality (%)**	3.6	18.4*	0.044
**Adverse Cardiac events (%)**			
**Myocardial Infarct (%)**	0	13.2*	0.022
**Arrhythmia's (%)**	19.6	50*	0.029
**Low Cardiac Output (%)**	1.8	60.5*	<0.001
**Wound infection (%)**	3.6	5.3	0.665
**Pneumonia (%)**	3.6	7.9	0.368
**Re-operation (%)**	8.9	5.3	0.773
**Duration of Mechanical Ventilation (h)**	9 (7)	38 (28)*	0.032
**Peak Serum Troponin (ng/L)**	1.7 (1.2)	12.3 (9.2)*	0.026
**Peak Serum Creatinin (mg/L)**	88 (24)	102 (46)	0.092
**Duration of stay in ICU (d)**	3.1 (1.5)	6.9 (5.5)*	<0.001
**Duration of stay in Hospital (d)**	10 (3)	14 (7)*	0.022

LV, left ventricular; CPB, cardiopulmonary bypass.

*P < 0.05, between the two groups; χ^2 ^test with Yates correction or unpaired Student *t *test.

**Figure 3:**
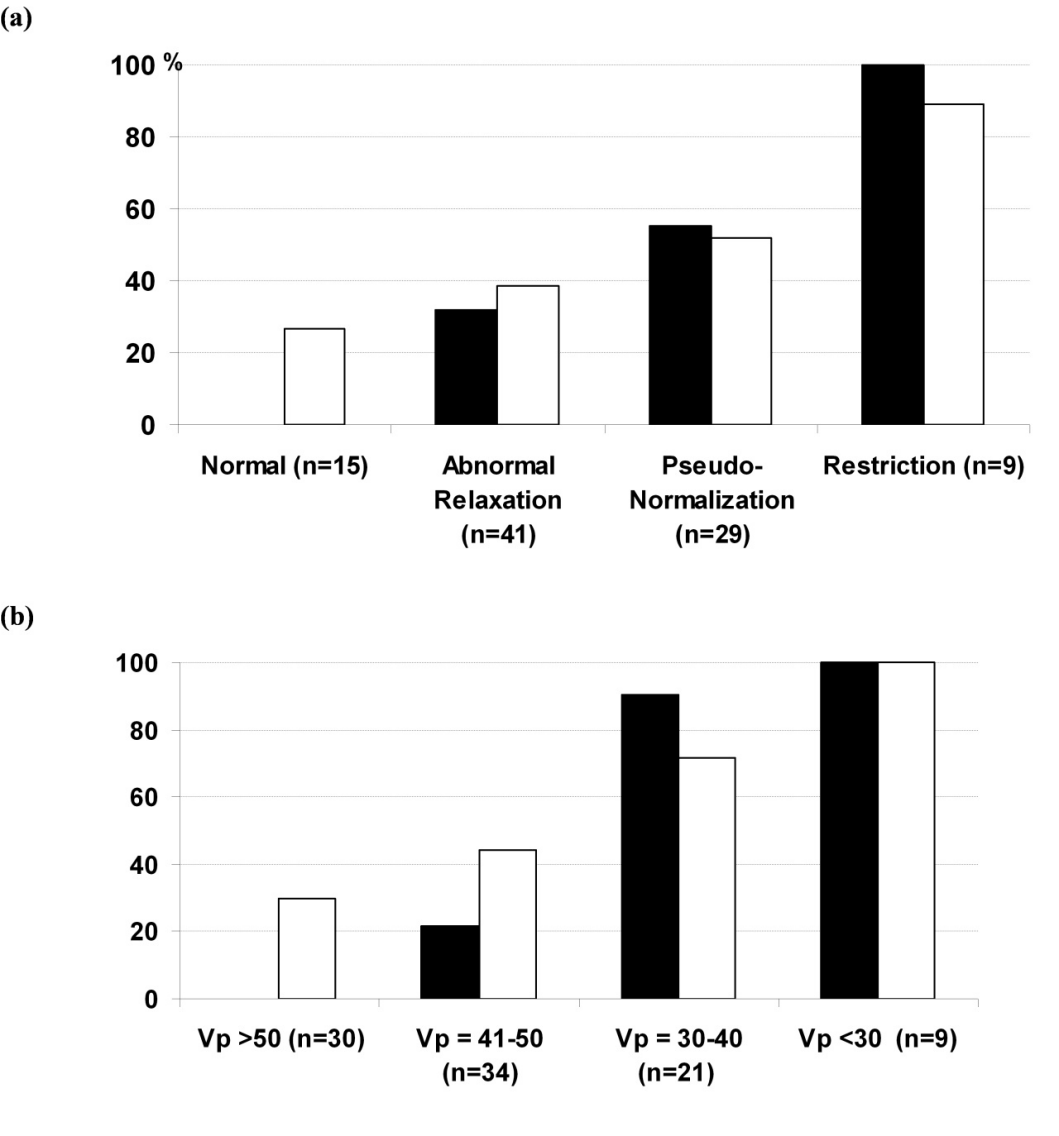
**Incidence of post-cardiopulmonary bypass left ventricular dysfunction (black square) and postoperative cardiac complications (open square**. Myocardial infarct, arrhythmias and/or low cardiac output syndrome) in relation with the severity of left ventricular diastolic dysfunction are expressed by standard classification **(a) **and by transmitral flow propagation velocity (Vp) **(b)**.

The incidence of LV dysfunction and cardiac complications increased significantly with the severity of diastolic dysfunction, particularly in patients with a restrictive filling pattern and those with Vp less than 40 cm/s (Figure 3). Noteworthy, LV dysfunction was observed in 28 out of 30 patients (90%) with low Vp (≤40 cm/sec) as opposed to 7 out of 64 patients with normal-to-high Vp.

## Discussion

In this prospective study, 40% of high-risk patients undergoing aortic valve replacement required inotropic support and/or an intraortic balloon pump for weaning from CPB. Advanced age, preoperative LV diastolic dysfunction and prolonged aortic clamping time were identified as independent risk factors of post-CPB LV dysfunction. Among the echocardiographic markers of LV diastolic dysfunction, the transmitral flow propagation wave (Vp) was found superior in terms of prognostic value and reliability. Below a cut-off value of 40 cm/s, 90% of patients required inotropic support after weaning from CPB as opposed to only 11% among those with preoperative Vp >40 cm/s.

The anesthetic and surgical techniques were all standardized and protocol-driven hemodynamic treatments were based on information gathered from pressure monitors and TEE examination. In contrast to previous large cohort studies, we focused on aortic valvular patients with an expected operative mortality ≥9% based on the Bernstein-Parsonnet algorithm [20]. The higher operative risk profile was mainly related to hypertension (84% of patients), advanced age (62% ≥70 years) hyperlipidemia (62%) and diabetus mellitus (28%), all factors known to participate in the development of LV hypertrophy and diastolic LV dysfunction [21].

Predictors of LV dysfunction after aortic valvular replacement have been investigated in four other studies which largely differ in their case-mix, hemodynamic treatments and criteria to define the main study endpoint [6-8,22]. In these cohort studies, inotropic therapy varied from 4% to 52% and was mainly related to advanced age, congestive heart failure, low LV ejection fraction, elevated LV end-diastolic pressure and prolonged aortic cross-clamping time. Interestingly, we found that patients with post-CPB LV dysfunction experienced higher plasma levels of troponin and a two-to-three fold increase in postoperative cardiac complications. Consistent with these data, Müller *et al*. reported a higher 30-day mortality rate among patients receiving inotropic drugs following cardiac surgery [22].

Our study is the first investigation assessing the prognostic implication of echocardiographic markers in addition to clinical and surgical variables in patients undergoing aortic valve replacement. Based on standard Doppler-derived measurements, more than 80% of patients presented LV diastolic dysfunction and, all of them had Vp <50 cm/s. This was consistent with previous reports identifying abnormal LV relaxation and filling patterns in more than 50% of elderly, in patients with aortic stenosis and those undergoing coronary artery bypass surgery [23,24]. As reported in longitudinal population-based studies, LV diastolic dysfunction often precedes the development of LV systolic impairment, conveying a poor prognosis, particularly after myocardial infarct, in congestive heart failure and in cardiac amyloidosis [25-27].

Preoperative LV diastolic dysfunction associated with myocardial hypertrophic and fibrotic changes could predispose patients to LV dysfunction during weaning from CPB for several reasons. First, patients with enlarged cardiac muscular mass and reduced capillary density are prone to develop ischemic lesions due to suboptimal delivery of the cardioplegic solution particularly after prolonged aortic cross-clamping time [28,29]. Second, accelerated apoptosis of hypertrophied cardiomyocytes may further decrease mechanical cardiac efficiency and has been shown to correlate with increased release of troponin following aortic valve surgery [30,31]. Third, LV diastolic dysfunction often coexists with latent or patent alterations in systolic LV function that corresponds to the clinical syndrome of *congestive heart failure *and the functional states of *elevated LV end-diastolic pressure *or *low LV ejection fraction *which are all considered strong predictors of LV dysfunction, cardiac complications and mortality after cardiac surgery [2,3,5-8,32].

Although Doppler-derived mitral inflow and pulmonary venous flow measurements as well as TDI currently provide the cornerstones of the assessment of LV diastolic function, their practical application in the operating room may be hampered by difficulties in recording and measuring each of these parameters within a short time in anesthetized cardiac patients. In addition, most of these echo-Doppler parameters are highly influenced by age, heart rate and loading conditions [33]. Therefore, dynamic tests such as the Valsalva manoeuvre are necessary to unmask impaired LV relaxation and to distinguish *pseudo-normalization *patterns. In our experience, these measurements were less reproducible (intra- and interobserver variabilities ranging from 7% to 10% and 8% to 12%, respectively) and could not be obtained in 13% of patients. In addition, extensive calcifications of the aortic valve likely restrain the downward excursion of the mitral annulus resulting in low peak annular velocities (E') which underestimates LV longitudinal relaxation. Likewise, the success rate of Doppler-derived pulmonary flow measurements has been reported within a wide range (37% to 99%) and with considerable inter-reader variability (3% to 21%) [34,35].

In agreement with other studies, we could easily determine Vp in all patients without post-acquisition manipulation and with minimal inter-and intraoperator variability (<5%) [34,36]. Basically, Vp reflects the spatio-temporal distribution of early diastolic blood flow generated by atrio-ventricular pressure gradients and vorticity resulting from shear between inflowing and stationary blood in the LV. A significant negative correlation has been demonstrated between Vp and the gold standard parameter of LV diastolic function, the time constant of relaxation (τ) [35]. Besides simplicity and reproductibility, Vp is less dependent on loading conditions and heart rate changes. Consistent with previous studies [24,37,38], below a threshold value of 50 cm/sec, Vp reliably detected all grades of diastolic dysfunction. In addition, analysis of ROC curves indicated that a cut-off value of 40 cm/sec was helpful to discriminate patients experiencing LV dysfunction after weaning from CPB that was also paralleled by an increased incidence of adverse cardiac events in the ICU. Likewise, Matyal *et al*. [39] confirmed the importance of LV diastolic dysfunction for risk stratification in vascular surgery. Below a Vp threshold of 45 cm/sec, patients were twice as likely to experience at least one postoperative adverse events than patients with Vp >45 cm. Taken together, these data suggest that, in the perioperative settings where hemodynamic conditions are changing often rapidly, Vp is better suited to evaluate LV diastolic function than the traditional echo-Doppler parameters.

We are mindful of several limitations. First, being conducted in a single centre with a relatively small population sample focusing mainly on LV function, this observational study requires further validation in a larger group of patients with a combined assessment of left and right ventricular function. Patients with arrhythmias and pulmonary hypertension were excluded from the study and the low prevalence of systolic LV failure, anemia and renal failure precluded any conclusion regarding these potential risk factors (type II error, false negative results). Second, Vp might underestimate the severity of diastolic dysfunction in cases presenting LV chamber dilation due to swirlings of the inflow along the LV wall [19,34]. Since less than 15% of our patients presented low LV ejection, we presume that low Vp values correctly reflect impairments in LV relaxation and filling. Third, although increased LV wall thickness was documented in all patients, we did not examine the influence of LV geometry (for example, excentric or concentric hypertrophy, remodelling) and plasma biomarkers of cardiac distension (for example, brain natriuretic peptides (BNP) on LV diastolic function. Interestingly, several reports have stressed the negative impact of concentric LV geometries (with or without enlarged cardiac mass) and of elevated BNP levels on in-hospital mortality and early cardiac complications [40-42].

## Conclusions

This study provides the first evidence that diastolic dysfunction as defined by Vp <40 cm/s, in addition to advanced age and prolonged ischemic time, identifies patients at risk of LV dysfunction after valvular aortic surgery. Clinicians should anticipate a greater impact of perioperative TEE to identify high-risk cardiac patients while improving fluid and inotropic/lusitropic drug treatments. The association of preoperative diastolic dysfunction with adverse cardiac outcome begs the question as to whether trials of specific perioperative strategies to improve LV relaxation and filling patterns should be considered in patients undergoing aortic valve surgery.

## Key messages

• Advanced age, preoperative LV diastolic dysfunction and prolonged aortic clamping time are significant predictors of LV dysfunction following CPB requiring inotropic support in patients undergoing valve replacement for aortic stenosis.

• Among several echocardiographic parameters, transmitral flow propagation velocity (Vp) less than 40 cm/sec best identified patients at higher risk of LV dysfunction after CPB and was associated with more frequent cardiac complications in the ICU.

## Abbreviations

BNP: brain natriuretic peptides; CI: confidence interval; CPB: cardiopulmonary bypass; DT: deceleration time; E' and A': early and late diastolic velocities of the mitral annulus; EDA: end-diastolic area; ESA: end-systolic area; FAC: fractional area change; IABP: intra-aortic balloon pump; IVRT: isovolumic relaxation time; LV: left ventricular; MAP: mean arterial pressure; ORs: odds ratios; PWT: posterior wall thickness; ROC: receiver-operator-characteristic; S: D and Ar: peak systolic, diastolic and atrial reversal velocities of pulmonary venous flow; TDI: tissue Doppler imaging; TEE: transoesophageal echocardiography; Vp: transmitral flow propagation velocity.

## Competing interests

The authors declare that they have no competing interests.

## Authors' contributions

ML and JD participated in the study design, data analysis, interpretation of the data as well as the writing of the manuscript. JD, VC and CI participated in data collection, literature search and data interpretation. AK, TC, TT and MC participated in revising the bibliography, and correcting and editing the manuscript. All the authors have read and approved the final manuscript.
